# Identification of risk factors associated with oral 5-aminolevulinic acid-induced hypotension in photodynamic diagnosis for non-muscle invasive bladder cancer: a multicenter retrospective study

**DOI:** 10.1186/s12885-021-08976-1

**Published:** 2021-11-13

**Authors:** Hideo Fukuhara, Takahiro Nohara, Koshiro Nishimoto, Yutaka Hatakeyama, Yuki Hyodo, Yoshiyasu Okuhara, Masafumi Oyama, Atsushi Mizokami, Keiji Inoue, Hideyasu Matsuyama, on behalf of the Japan Urological Photodynamic Society

**Affiliations:** 1 Department of Urology and Center for Photodynamic Medicine, Kochi Medical School, Kohasu, Oko, Nankoku, Kochi 783-8505 Japan; 2 Department of Integrative Cancer Therapy and Urology, Kanazawa University, 13-1, Takara-machi, Kanazawa, Ishikawa 920-8640 Japan; 3 Department of Urology, Saitama Medical University International Medical, Center1397-1, Yamane, Hidaka, Saitama 350-1298 Japan; 4 Center of Medical Information Science, Kochi Medical School, Kohasu, Oko, Nankoku, Kochi 783-8505 Japan; 5 Department of Urology, Graduate School of Medicine, Yamaguchi University, 1-1-1, Minami-kogushi, Ube, Yamaguchi 755-8505 Japan

**Keywords:** 5-aminolevulinic acid, Photodynamic diagnosis, Hypotension, Non-muscle invasive bladder cancer, Transurethral resection of bladder tumor

## Abstract

**Background:**

To investigate risk factors for orally administered 5-aminolevulinic acid (ALA)-induced hypotension for bladder cancer patients receiving photodynamic diagnosis (PDD)-assisted transurethral resection of bladder tumor (TURBT).

**Methods:**

Patients were categorized into two groups intraoperatively: a hypotensive group (minimum systolic blood pressure (SBP) ≤80 mmHg) and a non-hypotensive group (minimum SBP > 80 mmHg). We examined differences between the hypotensive group and non-hypotensive groups to identify clinical risk of ALA-induced hypotension using multivariate logistic regression analysis and decision tree analysis.

**Results:**

Among 282 cases with ALA-PDD-assisted TURBT from three institutions who were screened, 245 patients were included in the final analysis. In total, 156 patients (63.7%) showed any grade of hypotension during ALA-PDD-assisted TURBT. General anesthesia and spinal anesthesia were induced intraoperatively in 113 patients (46.1%) and 132 patients (53.9%), respectively. Median SBP at baseline (before taking ALA) and at the beginning of anesthesia was 127 mmHg (range, 69–186 mmHg) and 124 mmHg (range, 69–186 mmHg), respectively. Median minimum SBP during ALA-PDD-assisted TURBT was 75 mmHg (range, 43–140 mmHg). Multivariate logistic regression analysis revealed that history of hypertension (odds ratio (OR) 7.568, *p* < 0.05) and general anesthesia (OR 14.435, p < 0.05) as significantly associated with an increased risk of hypotension incidence. Use of calcium antagonist showed significant negative associations with hypotension (OR 0.183, *p* < 0.05). Decision tree analysis showed presence of general anesthesia, age ≥ 74 years and American Society of Anesthesiologists physical status (ASA-PS) ≥2 as the most important discriminators.

**Conclusions:**

General anesthesia and hypertension were independent risk factors related to ALA-induced hypotension. In contrast, use of calcium antagonists was identified as a factor associated with reduced risk of ALA-induced hypotension.

## Background

Accumulating evidence has suggested 5-aminolevulinic acid (ALA)-mediated photodynamic diagnosis (PDD) as a good method for diagnosing non-muscle invasive bladder cancer (NMIBC) [1]. Several randomized controlled trials have shown the feasibility of ALA-PDD for improving recurrence rates after transurethral resection of bladder tumor (TURBT) [2–6]. ALA-PDD enables to detect low visible lesions by white light source. ALA-PDD is thus an essential tool during TURBT for urologists.

Recently, ALA-PDD using the orally administrated ALA (ALAGRIO®) for NMIBC was approved for clinical use by the Pharmaceuticals and Medical Devices Agency and insurance coverage in 2017. A multicentre phase III study in Japan showed the feasibility of ALA-PDD for bladder cancer, offering 79.6% sensitivity and 80.6% specificity, respectively [7]. The clinical indication for ALAGRIO® is the visualization of NMIBC during TURBT. The recommended dose of ALA is 20 mg/kg body weight. The medical package insert for ALAGRIO® indicates that ALA-PDD should be started about 3 h after oral administration of ALA (range 2–4 h). ALA shows preferential uptake up by the liver, kidneys, endothelium and skin as well as tumors, and is metabolized to protoporphyrin IX (PpIX) in the mitochondria. The maximum PpIX plasma concentration is reached within 4 h after oral administration of ALA at the recommended dose, plasma levels then rapidly decline during the subsequent 20 h becoming undetectable after 48 h. ALA is eliminated efficiently with a half-life of 1–3 h. Approximately 30% of orally administrated ALA is excreted unchanged in urine within 12 h [8]. Patients taking oral ALA have experienced various transient adverse events of nausea, vomiting, photosensitivity, hypotension and liver enzyme dysfunctions. Previous studies have reported the incidence of ALA-induced hypotension as 2–3% [9, 10]. Cases of severe hypotension requiring intensive care management after using ALAGRIO® have been reported in Japan [11–13].

Chun et al. reported a history of hypotension and antihypertensive therapy as the only factor significantly associated hypotension after ALA administration with an odds ratio (OR) of 17.7 [14]. While 43% of our patients were on antihypertensive drugs, only 10% developed significant hypotension. However, Chun could not identify whether particular antihypertensive agents are responsible for hypotension. The mechanisms and clinical risk factors for ALA-induced hypotension after using ALAGRIO® have not been identified. This study retrospectively analyzed the incidence and risk factors of ALA-induced hypotension using ALAGRIO®.

## Methods

### Study cohort

This multi-center retrospective study included clinical data from 282 consecutive bladder cancer patients who underwent ALA-PDD-assisted TURBT at Kochi Medical School Hospital, Saitama Medical University Hospital, or the Kanazawa University Hospital in Japan between December 2017 and April 2019. Patients in whom systolic blood pressure was not measured during the operation were excluded from analysis.

ALAGRIO (20 mg/kg) was dissolved in 50 mL of normal saline, then an aqueous solution of ALAGRIO was orally administrated before surgery. ALA-PDD was started around 3 h after oral administration (range 2–4 h). Blood pressure was measured before and after taking ALAGRIO in all patients who underwent ALA-PDD-assisted TURBT.

After the patient was administrated to the operating room, standard monitoring, namely, electrocardiography, non-invasive blood pressure measurement, and pulse oximetry was performed. Individual anesthesiologists decide on the anesthetic procedure in accordance with the patient’s condition. An arterial catheter was inserted into the radial artery for continuous blood pressure monitoring by individual anesthesiologists’ decision. Vital signs including blood pressure were also measured over time during ALA-PDD-assisted TURBT. Vasopressor was administered appropriately according to the appearance of hypotension during the operation.

The aim of this study was to examine the risk of hypotension during ALA-PDD-assisted TURBT in a multicenter study. The three institutions of Kochi medical school hospital, Kanazawa university hospital and Saitama medical university international medical center participated in this study. The Ethics committee of Kochi medical School has waived the need of informed consent for this study because of retrospective nature of the study (approval no.31–88). Data were obtained from all patients who completed a general consent form based on the opt-out policy of each of the three institutions. This study was conducted in accordance with the ethical principles for medical research on human subjects, including research into identifiable human materials and data, as stated in the Declaration of Helsinki (64th World Medical Association General Assembly, Fortalenza, Brazil, October 2013).

### Definition of hypotension

Hypotension during ALA-PDD-assisted TURBT was diagnosed using the following criteria: minimum systolic blood pressure (SBP) ≤80 mmHg one or more times after induction of anesthesia according to the criteria described by Wesselink et al. [15]. The primary outcome was the incidence of hypotension. All patients were categorized into one of two groups in this study: a hypotensive group, comprising patients who showed intraoperative SBP ≤ 80 mmHg; and a non-hypotensive group of patients who never showed intraoperative SBP ≤80 mmHg. This study examined the differences between hypotensive and non-hypotensive groups to identify clinical risk factors for ALA-induced hypotension.

### Data collection

This retrospective study used data obtained from the clinical database of Kochi medical school hospital, Kanazawa university hospital and Saitama medical university international medical center. Multivariate logistic regression analysis were used with the following variable as covariates: age, sex, liver function, estimated glomerular filtration rate (eGFR), American Society of Anesthesiologists physical status (ASA-PS), anesthesia type (general or spinal), SBP before oral administration of ALAGRIO, SBP on entry to the operating room, SBP at start of anesthesia, and perioperative minimum SBP. To determine risk factors for hypotension, we also investigated comorbidities of atrial fibrillation (Af), paroxysmal Af (pAf), cardiovascular disease, hypertension and treatment by Calcium antagonists (Ca-blockers), angiotensin II receptor blockers and other antihypertensive drugs.

### Statistical analysis

Hypotensive and non-hypotensive groups were compared to identify risk factors for ALA-induced hypotension by multivariate logistic regression analysis and decision tree analysis. Normally distributed data are expressed as median and interquartile range, whereas percentages are used for continuous variables. Differences between groups were assessed using the Mann-Whitney U test and the chi-squared test, and value of *p* < 0.05 were considered statistically significant. The Bonferroni method was used to adjust *p*-values for multiple comparisons.

Multivariate logistic regression analysis was performed to independent associations between each covariate and frequency of hypotension. Adjusted ORs and 95% confidence intervals (CIs) were calculated using epiDisplay software (Epidemiological Data Display Package), with values of *p* < 0.05 considered statistically significant. Statistical analysis was performed using R software (version 4.0.2; R Foundation for Statistical Computing, Vienna, Austria).

Decision trees analysis is a nonparametric supervised learning model. This form of analysis does not require the explanatory variables to be linear for model generation, and outliers are considered automatically. Training of the decision tree is done by splitting the original set into subsets based on the values of the explanatory variables. This process is repeated recursively for all subsets. Nodes close to the root node, representing the top of the resulting tree model, are important for classification. Decision trees analysis was used as explanatory model aimed at confirming the results of multivariate logistic regression analysis and estimating risk factor thresholds. The tree model was calculated using the CART algorithm in the rpart package (Recursive Partitioning and Regression Trees. R package version 4.1–15, Vienna Austria).

## Results

**Fig. 1:**
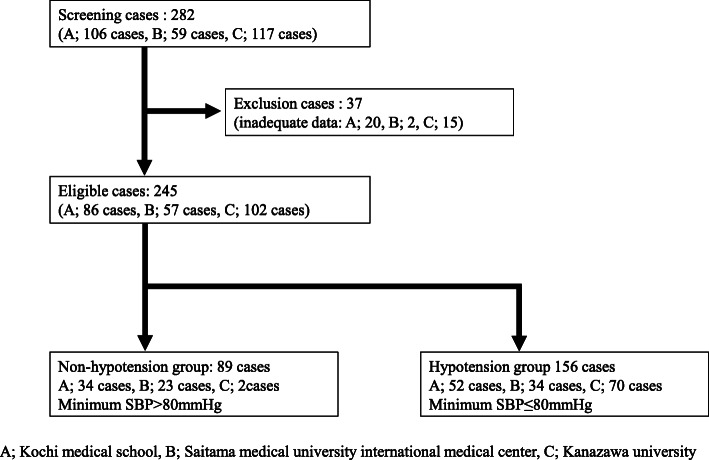
Flow chart of the study cohort for the entire study period. A: Kochi Medical School; B: Saitama medical university international medical center; C: Kanazawa university

A flow chart of the study population is shown in Fig. 1. In this study, 282 cases were first enrolled, then 37 cases were excluded based on the protocol. A final total of 245 cases were selected for analysis, comparing 89 cases in the non-hypotensive group and 156 cases in the hypotensive group (Table 1). Baseline characteristics of all patients are summarized in Table 1. The frequency of hypotension for the total cohort was 63.7% (95%CI 57.3–69.7%), and median age was 75 years (range 47–92 years). General anesthesia and spinal anesthesia were induced for 113 patients (46.1%) and 132 patients (53.9%), respectively. Median SBPs at baseline (before taking ALAGRIO) and at start of anesthesia were 127 mmHg (range 69–186 mmHg) and 124 mmHg (range 69–186 mmHg), respectively. Median minimum SBP during ALA-PDD-assisted TURBT was 75 mmHg (range 43–140 mmHg). In the hypotension group, median SBPs at baseline and at start of anesthesia were 126 mmHg (range 71–175 mmHg) and 115 mmHg (range 70–180 mmHg), respectively. Median minimum SBP during ALA-PDD-assisted TURBT was 70 mmHg (range 43–79 mmHg). In the non-hypotensive group, median SBPs at baseline and at start of anesthesia were 130 mmHg (range 100–182 mmHg) and 130.5 mmHg (range 90–180 mmHg), respectively. Median minimum SBP during ALA-PDD-assisted TURBT was 90.5 mmHg (range 80–140 mmHg).

**Table 1:**
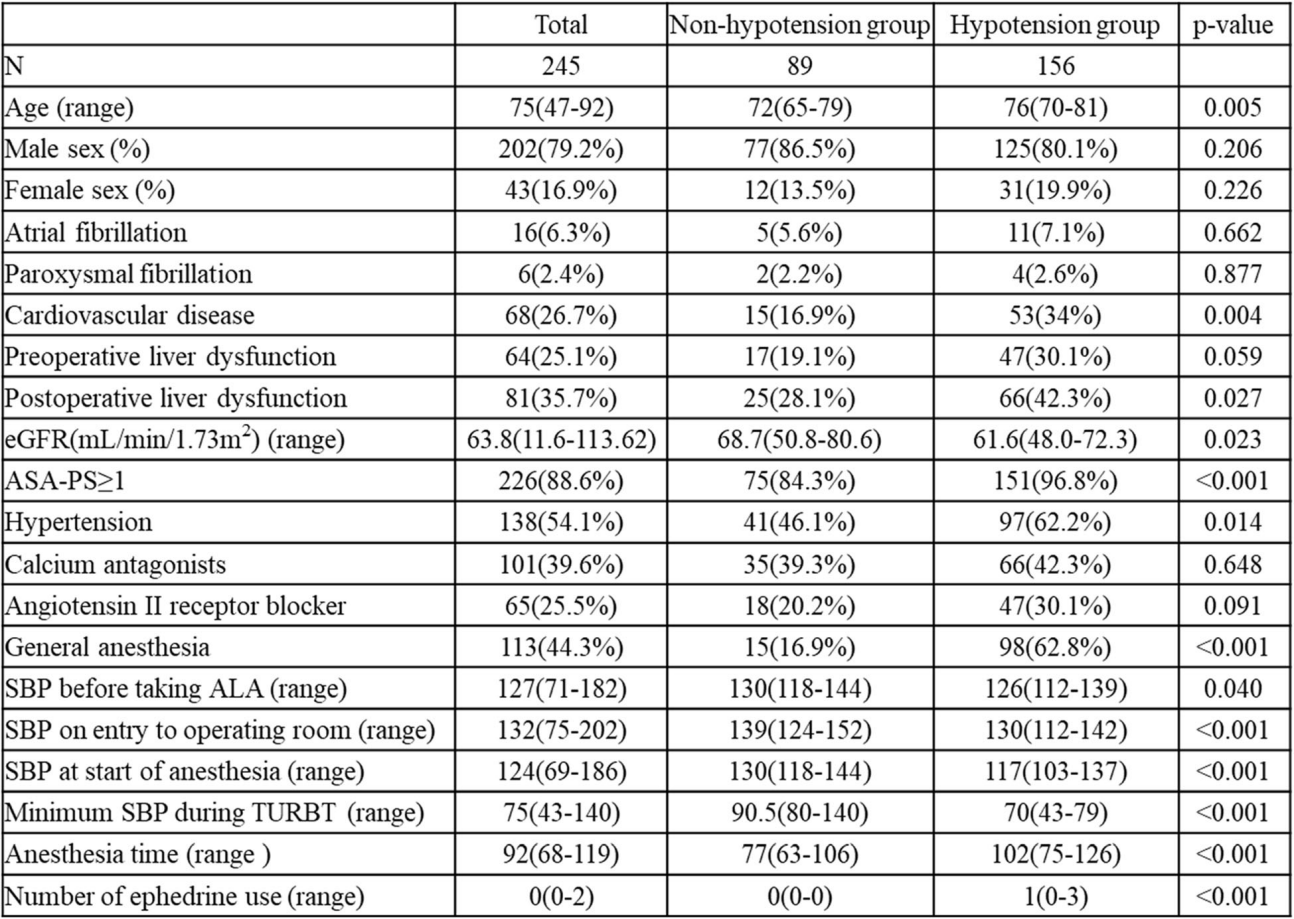
Characteristics of the 245 patients included in final analysis. Numerical variables are shown as median and range. Hypotension is diagnosed as minimum systolic blood pressure (SBP) ≤80 mmHg. Values of *p* < 0.05 are considered to indicate a significant difference between the non-hypotension group and the hypotension group

Table 2 shows adjusted ORs for event estimates of all patients from multivariate logistic regression analysis. Hypertension, use of calcium antagonists, and general anesthesia showed statistical significance. Histories of hypertension (OR 7.568, *p* < 0.05) and general anesthesia (OR 14.435, *p* < 0.05) were significantly associated with an increased risk of hypotension. History of calcium antagonists use showed a significant negative association with hypotension (OR 0.183, *p* < 0.05).

**Table 2:**
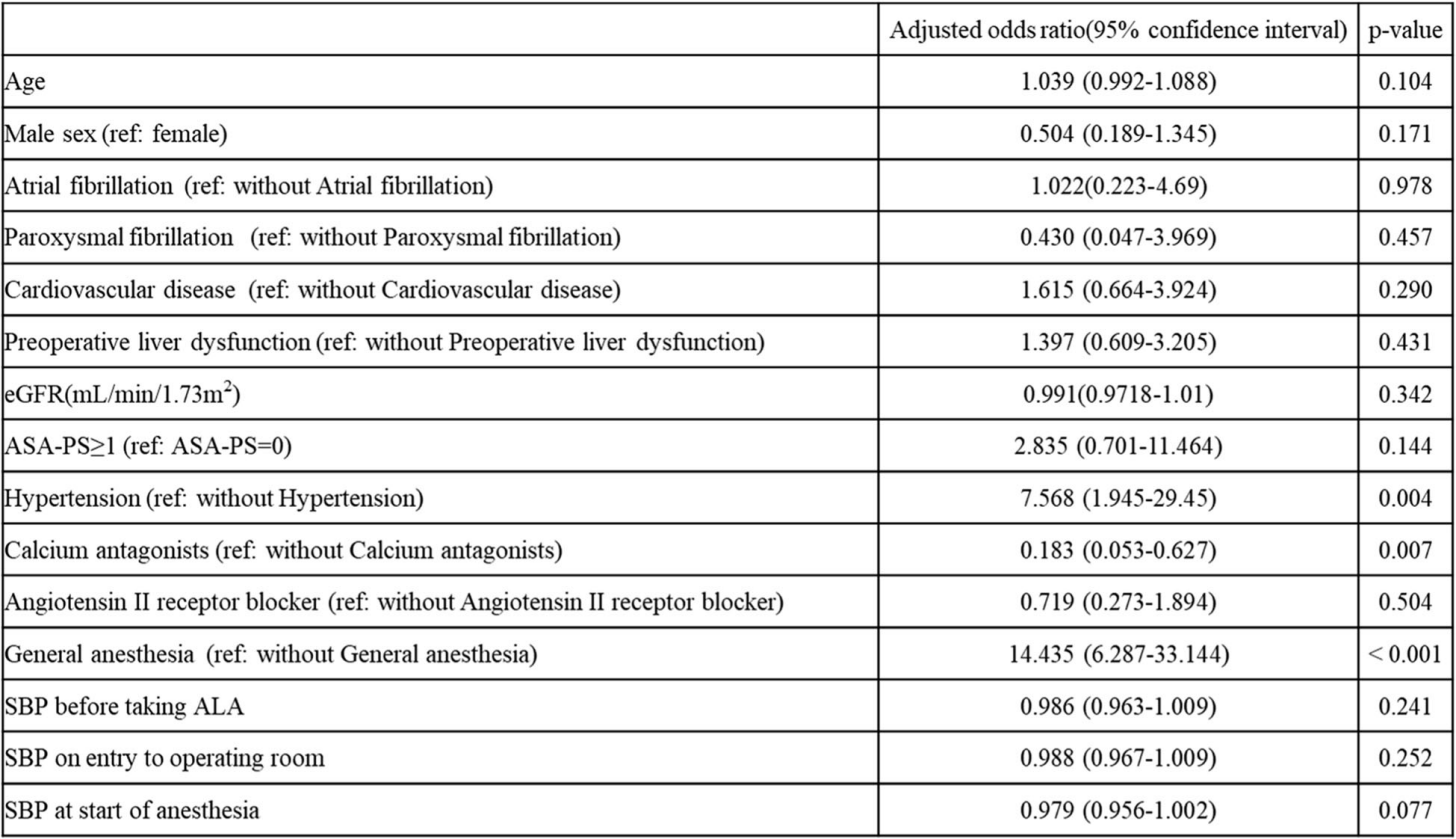
Multivariate logistic regression analysis between the study groups to identify risk factors for ALA-induced hypotension. Adjusted odds ratios (ORs) and 95% confidence intervals (95%CIs) were shown Values of *p* < 0.05 are considered statistically significant

**Fig. 2:**
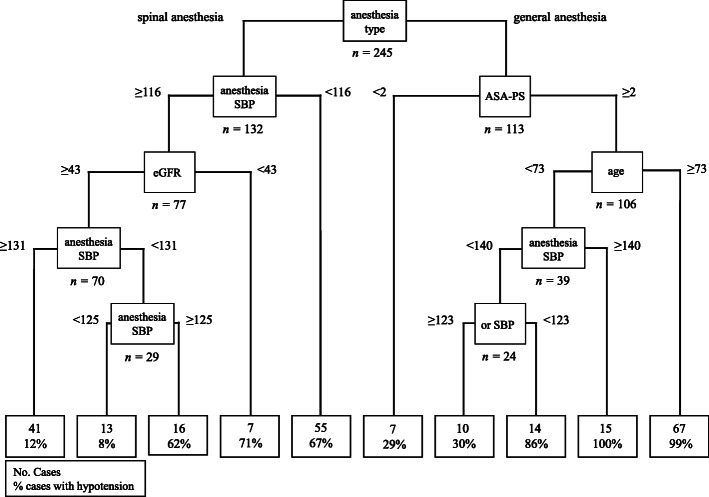
Decision tree model for predicting hypotension. Branches of the explanatory variables employed in the decision tree model are called nodes. Each node shows the variables and thresholds, sample size after the bifurcation, and the bottom node shows the number and percentage of participants with hypotension. Anesthesia SBP: SBP at the beginning of anesthesia; eGFR: estimated glomerular filtration rate; OR SBP: SBP on entry to operating room

The final decision tree had four tiers of branching using eight categorical variables, resulting in ten terminal nodes. Figure 2 shows a decision tree model for predicting hypotension. Anesthesia type and ASA-PS and SBP at start of anesthesia were important predictors of hypotension. In particular, the decision tree model showed that predictors of hypotension differed by anesthesia type. Patients with general anesthesia exhibited a higher frequency of hypotension among patients with ASA-PS of 2–3 and age > 73 years. In patients with spinal anesthesia, the frequency of hypotension increased with SBP at start of anesthesia < 116 mmHg, and decreased with eGFR > 43 mL/min/1.73m^2^ and SBP at start of anesthesia > 131 mmHg.

In summary, the presence of general anesthesia, age ≥ 74 and ASA ≥2 was the most important discriminators; 99% of all patients experienced hypotension meeting these 3 criteria. The presence of SBP ≤115 mmHg at the beginning of anesthesia under spinal anesthesia was the second discriminator; 67% of all these patients experienced hypotension. In patients without SBP ≤115 mmHg at the start of spinal anesthesia, the presence of eGFR ≤42 ml/min/1.73m^2^ was the third discriminator; 71% of all such patients experienced hypotension. AE grades of hypotension according to CTCAE v4.0 were as follows: Grade 1, 40cases; Grade 2, 28 cases; Grade 3, 35 cases; Grade 4, 5 cases. All patients with grade 4 hypotension needed continuous administration of adrenaline during operation, and 4 grade 4 cases needed intensive care management postoperatively. All grade 4 cases showed complete improvement of hypotension by the next day. No cases required extended hospitalization or showed remaining aftereffects of ALA-induced hypotension. Among all patients, 48 (19.6%) cases experienced liver dysfunction after TURBT. Liver dysfunction was transient and gradually normalized without any treatment.

## Discussion

Patients taking oral administration of ALA have experienced various transient adverse events compared to those receiving intravesical administration of ALA [7, 9, 12, 14]. Peak plasma concentrations of ALA were achieved about 1 h after oral administration [16]. Plasma ALA concentrations declined with a terminal half-life of approximately 45 min. The rapid appearance of ALA in plasma after oral administration suggests that ALA is rapidly absorbed via this route [17, 18]. Approximately 60% of ALA was absorbed from the intestine after oral administration, and about 30% of orally administrated ALA was excreted as unchanged drug in the urine [19]. The AUC of ALA in plasma was about 50-fold higher than that for PpIX in plasma after oral administration of ALA. The PpIX level in plasma showed no significant difference between intravenous and oral administrations of ALA. Dalton JT et al. indicated that oral administration of ALA provides a degree of systemic PpIX accumulation equivalent to that with intravenous administration [8].

Less than 1% of the intravesical dose was absorbed from the bladder in intravesical ALA administration [20]. The ratio of AUC in urine to AUC in plasma was 20,000-fold greater AUC of the bladder compared with plasma [20]. The bladder was exposed to significantly higher concentrations of ALA than plasma following intravesical ALA administration. The marked differences in plasma concentrations of ALA and PpIX between oral and intravesical administration might be related to differences in the occurrence of hypotension.

Intraoperative hypotension is a well-known adverse event in general anesthesia. Wesselink et al. investigated the association between intraoperative hypotension and postoperative outcomes. They indicated that prolonged exposure (> 10 min) to SBP < 80 mmHg and even short durations of exposure to < 70 mmHg were associated with mildly elevated risk of any end-organ surgery [15]. In addition, they suggested 6 adverse outcomes related to intraoperative hypotension: mortality, acute kidney injury, myocardial injury, stroke, delirium and length of hospital stay. The use of vasopressors and fluid management were suggested to prevent postoperative organ dysfunction [15].

Nohara et al. reported that the frequency of vasopressor use was significantly higher during ALA-PDD-assisted TURBT compared to conventional TURBT [12]. On multivariate analysis, they indicated that general anesthesia and regular use of renin-angiotensin system inhibitors were associated with an increased risk of ALA-induced hypotension [12]. Minimum intraoperative SBP using renin-angiotensin system inhibitors was significantly lower than that in patients not using such agents. Chung et al. reported intraoperative hypotension with oral ALA administration in human malignant glioma [14]. They defined two groups based on ALA dose: group1, comprising patients who received ALA dose ≤20 mg/kg; and Group 2, comprising patients who received ALA dose > 20 mg/kg. Hypotension was seen in 5 of 44 patients (11%) in Group 1 and 5 of 46 patients (11%) in Group 2, showing no significant difference. In multivariate regression analysis, they showed that the only significant factors for ALA-induced hypotension were history of hypertension and antihypertensive use with an OR of 17.7 (*p* < 0.05). However, Chung et al. could not identify whether any particular antihypertensive drugs were responsible for hypotension.

This study identified general anesthesia and hypertension as independent risk factors related to ALA-induced hypotension in multivariate logistic regression analysis. In contrast, calcium antagonists were identified as factors that reduced the risk of ALA-induced hypotension. Although previous reports have described general anesthesia and hypertension as risk factors, this is the first study to identify calcium antagonists as reducing the risk. Decision tree analysis revealed a combination of multiple factors in a prediction model related to ALA-induced hypotension, as follows. First, general anesthesia, age ≥ 74 years and ASA-PS ≥ 2; second, SBP ≤115 mmHg at the beginning of anesthesia under spinal anesthesia; and third, SBP ≥115 mmHg at the beginning of anesthesia under spinal anesthesia and e-GFR ≤42 ml/min/1.73m^2^ .

Mingone et al. showed data consistent with increasing PpIX biosynthesis from ALA, resulting in soluble guanylate cyclase (sGC) activation and a cyclic guanosine monophosphate (cGMP)-elicited increase in animal studies [21]. As a lyase enzyme, sGC converts guanosine triphosphate (GTP) to cGMP, and cGMP plays an important role in smooth muscle relaxation, including the regulation of vasodilation. This mechanism may be one of contributors to decreases in systemic artery pressure among patients treated with ALA. The detailed mechanisms of the interactions between ALA and the two factors of general anesthesia and calcium antagonists should be revealed by basic research in the future. Our study had the following limitations. Firstly, because we evaluated the relation between systolic blood and ALA, our study did not evaluate that between diastolic blood pressure and ALA. The aim of this study was to identify risk factors of ALA-induced hypotension. Secondly, our cohort study did not examine an additional validation. So, further validation examination is necessary by other cohort studies.

In conclusion, the present study identified the independent risk factors and a prediction model using a combination of multiple factors related to ALA-induced hypotension. In clinical settings, ALA-induced hypotension is an important adverse event that is solvable.

## Data Availability

The datasets used and/or analyzed during the current study available from the corresponding author on reasonable request.

## Abbreviations

ALA: 5-aminolevulinic acid
PDD: Photodynamic diagnosis
SBP: Systolic blood pressure
OR: Odds ratio
ASA-PS: American Society of Anesthesiologists physical status
NMIBC: Non-muscle invasive bladder cancer
eGFR: Estimated glomerular filtration rate
Af: Atrial fibrillation
pAf: Paroxysmal Af
CIs: Confidence intervals
sGC: Soluble guanylate cyclase
cGMP: Cyclic guanosine monophosphate
GTP: Guanosine triphosphate
